# T cell epitope content comparison using EpiCC correlates with vaccine efficacy against heterologous porcine reproductive and respiratory syndrome virus type 2 strains

**DOI:** 10.3389/fmicb.2025.1625309

**Published:** 2025-06-26

**Authors:** J. Mark Hammer, Andres H. Gutierrez, Lucas Huntimer, Benjamin Gabriel, William D. Martin, Sabine E. Hammer, Tobias Käser, Anne S. De Groot

**Affiliations:** ^1^Elanco Animal Health, Greenfield, IN, United States; ^2^EpiVax Inc., Providence, RI, United States; ^3^GenVax, Ames, IA, United States; ^4^Department of Biological Sciences and Pathobiology, University of Veterinary Medicine Vienna, Vienna, Austria

**Keywords:** porcine reproductive and respiratory syndrome virus (PRRSV), T cell epitope, vaccine, challenge, immune response, T cell response

## Abstract

Porcine reproductive and respiratory syndrome virus (PRRSV) is one of the most ubiquitous RNA viruses affecting pigs and pig farms globally. While vaccines are available, they are not entirely effective, and the introduction of modified live virus vaccines (MLV) has contributed to an increase in the rate of viral evolution. While vaccines induce humoral responses that may contribute to immunity against PRRSV, vaccines containing T cell epitopes that are well-matched to circulating strains are believed to be more likely to induce protective effects upon challenge or field exposure. We developed an algorithm that performs T cell epitope content comparison (EpiCC) based on PRRSV sequence data, that may assist veterinarians, practitioners, producers, and farmers to select and design well-matched vaccines for use against circulating PRRSV isolates. A recently published vaccination-challenge experiment provided an opportunity to test EpiCC. We hypothesized that higher conservation of T cell epitope content between the MLV vaccine and challenge viruses would be associated with better protective effects of vaccination. We used the EpiCC algorithm to compare the T cell epitope content contained in the MLV Prevacent^®^ vaccine used in the study and four heterologous type 2 (PRRSV-2) challenge strains. In this comparison, higher EpiCC coverage scores correlated not only with higher T cell responses observed in the efficacy study but also with better protection. The results also indicate that while genotyping may currently depend on GP5 analysis, it is unlikely that the genotyping performed using GP5 will be closely associated with protective relationships between vaccines and lineages. This suggests T cell epitope analysis of existing and new vaccines for epitope coverage may improve vaccine selection for an economically important porcine virus; it also points to the need to measure and thus improve T cell epitope content in PRRSV vaccines to maximize their protective efficacy against field strains.

## Introduction

Porcine reproductive and respiratory syndrome virus -type 2 (PRRSV-2) is the costliest endemic disease affecting swine in North America (Holtkamp et al., 2012; Holtkamp et al., 2013), with ∼30% of breeding farms experiencing active PRRSV-2 circulation annually (Paploski et al., 2021). Experimental work and field observations have repeatedly shown that immunization protocols (via modified live vaccines or killed whole virus inoculation) have only limited efficacy in preventing PRRSV-2 infection and transmission (Cui et al., 2024). PRRSV-2 evolutionary and epidemiological dynamics are characterized by the routine emergence and spread of novel genetic variants (Tang et al., 2025). Phylogenetic studies have demonstrated that genetic differences occur at key B cell epitopes, supporting the hypothesis that evolution of the lineages may be due, in part, to escape from humoral immunity (Paploski et al., 2019; Paploski et al., 2021).

Furthermore, immune-mediated selection is thought to be a key driver in the rapid evolution and antigenic diversification of PRRSV-2. The extent to which T-cell epitope diversity contributes to immune escape and antigenic divergence between lineages has only recently been subjected to study (Baker et al., 2025). T cell epitope analysis of 30 years of PRRSV-2 sequence data supported the hypothesis that PRRSV lineages are immunologically distinct, and provided new evidence that existing modified live virus (MLV) vaccines may not be well matched to current wild-type strains, at least from the perspective of adaptive immunity (T cell epitopes). However, evidence that T cell epitope conservation between vaccine strains and field strains promotes protective efficacy has been lacking. This has been due in part to the paucity of sequence data available for structural proteins other than GP5 in the PRRSV-2 genome databases and the lack of information on the heterologous immunogenicity and efficacy of individual vaccines in challenge studies. One such study was recently published (Proctor et al., 2022), making it possible to apply newly developed tools to evaluate the impact of T cell conservation between vaccine and challenge virus, for four PRRSV-2 field strains.

Following the publication of the study by Proctor et al. (2022), in which the authors showed variable efficacy of a vaccine against four challenge strains, we formulated the hypothesis that examination of the conservation of the T cell epitopes found in the live virus in the MLV vaccine and the T cell epitope content of the challenge strains might explain the observed differences in protection against field strains. The publication provided detailed results on heterologous vaccine immunogenicity (including IFN-γ production, T-cell proliferation and differentiation analysis as well as antibody analyses) and vaccine efficacy (e.g., viral loads, and lung pathology, and temperature data). This detailed analysis expanded the number of potential comparisons that could be performed to define the relationship between shared T cell epitope content and clinical outcomes. Correlation analyses for this study revealed that non-PRRSV strain-specific serum IgG levels and PRRSV strain-specific CD4 T-cell response were the best immune correlates of protection.

The computational method employed in the aforementioned 30 year retrospective and the current analysis is called T cell “Epitope Content Comparison” (EpiCC). The EpiCC algorithm evaluates similar sequences (vaccines and challenge strains or vaccines and field strains) as a means of evaluating the potential for T cell epitope cross-conservation to contribute to protective immunity. The analysis begins by applying another computer-based tool, PigMatrix, to define the putative class I and class II Swine Leucocyte Antigen (SLA) T-cell epitopes present in the vaccine and field strain sequences (Gutiérrez et al., 2015). Genetic sequences encoding that protein are then analyzed using the EpiCC algorithm, which assesses how many of the putative epitopes would induce T cell responses (or cell-mediated immunity) after vaccination and, when comparing sequences to the field strain protein sequences, how many T cell epitopes would be held in common. The latter information can be expressed as an EpiCC score, to describe the degree of antigenic relatedness that may be relevant to T cell responses (Gutiérrez et al., 2017). Higher EpiCC coverage scores suggest greater relatedness and higher T cell epitope similarity between the field strain and the vaccine.

In addition to generating EpiCC scores that reflect conserved T cell epitope content, the vaccine to field strain relatedness can be quantified as vaccine “coverage” of field strain T cell epitope content. Coverage quantifies how much of the field strain’s T cell epitope content is represented in the vaccine. T cell epitope coverage is calculated by dividing the EpiCC score calculated for each vaccine-strain pair by the total T cell epitope content of the respective field strain. Higher coverage suggests a potential for better protection. EpiCC coverage values can help to identify vaccines that may confer the broadest cross-reactive immunity and protection, not only for PRRSV, but for other swine pathogens for which cell-mediated immunity is relevant (Bandrick et al., 2020; Gutiérrez et al., 2017).

Here, we evaluated the seven structural proteins contained in the MLV vaccine strain for T cell epitopes and compared the vaccine epitopes to epitopes identified in the same proteins from the four wildtype PRRSV-2 challenge strains (VR2332, NADC20, NADC30, and NC174) using the PigMatrix and the EpiCC tools in the iVAX toolkit (Gutiérrez et al., 2015; Moise et al., 2020). When compared to the results of the Proctor et al. PRRSV challenge study, higher EpiCC scores (reflecting broader T cell epitope coverage) were indeed correlated with partial protection in the efficacy study.

This study suggests that EpiCC may complement current methods for determining vaccine-to-strain relatedness for PRRSV-2. Using EpiCC, a numerical threshold of protective efficacy for PRSSV2 vaccine was identified in the present study. Although the present study would need to be expanded to increase confidence in these estimated thresholds before applying that threshold more widely to other vaccines or field strains. The approach may also be helpful to determine which of the viral antigens are more likely to be associated with protection. In addition, EpiCC is likely to be useful for vaccine-to-strain comparisons for other swine and human viruses beyond PRRSV.

## Materials and methods

### Vaccine and challenge strain and protein information

The amino acid sequences of the Prevacent vaccine strain (MLV vaccine) and the sequences of the four challenge strains (VR2332, NADC20, NADC30, and NC174) were provided by the authors JMH and TK. For each strain, seven structural proteins (E, GP2, GP3, GP4, GP5, M, and N) were evaluated. The ectodomain of GP5 (30–60) was analyzed as an individual protein.

### Study population

The original study (Proctor et al., 2022) involved vaccinating the pigs either with mock or the MLV vaccine (30 pigs each). Four weeks post vaccination, six mock and six MLV vaccinated pigs each received one of five challenge inoculations—mock, VR2332, NADC20, NADC30, and NC174 (six unvaccinated and six vaccinated animals per challenge group). Table 1 shows the number of animals per challenge group in the vaccination study.

**TABLE 1:** Immune responses of study population.

Challenge virus	Number of animals	CD4 IFN-γ response (14 DPC)^a^			Low responders with inferred SLA-DRB1 alleles	High responders with inferred SLA-DRB1 alleles	Animals with available prediction model	SLA-DRB1 alleles used for prediction (response)
		Low (< 10%)	Inter-mediate	High (> 25%)				
VR2332	12	8	4	0	1	0	1	05:01 (low)
NADC20	12	3	3	6	0	2	2	02:01, 07:01 (high), 02:01 (high)
NADC30	12	5	4	3	1	0	0	–
NC174	12	6	4	2	3	0	1	02:01 (low)
Mock	12	12	0	0	0	0	0	–
Total	60	34	15	11	5	2	4	4

^a^14 days post challenge response measured by flow cytometry. Response level was defined based on the % IFN-γ+ within CD4 T cells.

Using data from the original PRRSV-2 study (Proctor et al., 2022), immune responses were defined based on the CD4 IFN-γ responses at 14 days post challenge (DPC), which represents 42 days post vaccination. Information derived from this study on the individual pig viral load, lung lesions, and T cell responses is provided later in the “Results” section. Low and high responders that had information available on their SLA-DRB1 alleles that matched available SLA-DRB1 T cell epitope prediction models are identified in Table 1. SLA-DRB1 information was obtained after the study was performed.

### Population-level and individual pig analysis

The first phase of this analysis involved a population-level comparison between the T cell epitope content of seven proteins (E, GP2, GP3, GP4, GP5, M, and N) from MLV vaccine to that of four wild-type challenge PRRSV strains (VR2332, NADC20, NADC30, and NC174). Here, population-level refers to the inclusion of SLA alleles that are believed to cover most types of genetic backgrounds thought to be represented by pigs that were included in the study. This analysis included T cell epitope predictions for both SLA class I and class II alleles to define the conservation of individual T cell epitopes contained in the vaccine sequences and the sequences in the field strains, as shown below. Next, evaluation of class I and class II T cell epitope conservation between the vaccine and the challenge strain used in individual pigs was performed.

### Prediction of T cell epitopes (population-level)

PigMatrix analysis (population level): To ensure an accurate prediction of immunogenic potentials, it is important to evaluate vaccine candidates and field strains for epitope content restricted by SLA that are commonly expressed in outbred populations. The distribution of SLA alleles among pig herds in the United States is unknown. As a first proxy for commonly expressed alleles, a set of SLA class I and II alleles that were frequently expressed in a cohort of pigs tested in a previous study were selected for this study (Gutiérrez et al., 2016). The alleles selected to represent SLA class I are SLA- 1*08:01, SLA- 1*12:01, SLA- 1*13:01, SLA- 2*05:01, SLA- 2*12:01, SLA- 3*05:01, SLA-3*06:01, and SLA-3*07:01. Additional available T cell epitope predictions for SLA-1*01:01, SLA-1*04:01, SLA-1*02:01, SLA-2*04:01, and SLA-3*04:01 were also included. The alleles selected to represent SLA class II are SLA- DRB1*02:01, *04:02, *06:02, *07:01, and *10:01. Additional available T cell epitope predictions for SLA- DRB1*01:01, *04:01, *06:01 were also included. PigMatrix prediction matrices were developed using the pocket profile method (Sturniolo et al., 1999) and well-defined EpiMatrix binding preferences for human Major Histocompatibility Complex (MHC) pockets (Gutiérrez et al., 2015). The pocket profile method uses sequence and structural similarities between swine and human major histocompatibility complexes to infer SLA peptide binding preferences leveraging available human information (Sturniolo et al., 1999).

Each of the structural protein input sequences were parsed into overlapping 9-mer frames and each frame was evaluated with respect to all the selected class I and class II SLA listed above. PigMatrix assessment scores range from approximately −3 to +3 for each of the SLA and are normally distributed. PigMatrix assessment scores above 1.64 are defined as “hits”; that is to say, potentially immunogenic and worthy of further consideration. Given a random peptide set, we expect about 5% of all assessments to score above 1.64. These peptides have a significant chance of binding to SLA molecules with moderate to high affinity and, therefore, have a significant chance of being presented on the surface of antigen presenting cells such as dendritic cells or macrophages where they may be interrogated by passing T cells. Once obtained, the information for each of the strain-specific structural proteins was then compared to the T cell epitope content of the structural proteins in the vaccine using the EpiCC tool.

### Prediction of T cell epitopes for individual pigs

PigMatrix analysis (individual pigs): To perform animal-specific T cell epitope content comparisons, we limited the comparisons to pigs for which available SLA-DRB1 prediction models were available, and in some cases, developed new models for individual animals. SLA-DRB1 alleles were inferred from low-resolution SLA class II haplotypes, based on published data and expert input. PigMatrix prediction matrices were developed using the pocket profile method (Sturniolo et al., 1999) and well-defined EpiMatrix binding preferences for human MHC pockets (Gutiérrez et al., 2015). Three of the low responders with inferred SLA-DRB1 alleles that were challenged with NADC30 and NC174 were not analyzed because they expressed an allele for which we were not able to develop a prediction model. This was due to low pocket similarity between swine and human MHC for these alleles.

To perform the analysis, the input sequences (amino acid sequence for each protein) for the vaccine and the challenge strains were parsed into overlapping 9-mer frames. The sequence in each frame was evaluated for binding potential with respect to the individual, animal-specific, SLA-DRB1 allele(s). This information was then compared to the T cell epitope content of the vaccine using the EpiCC tool.

### EpiCC analysis

EpiCC assesses the relatedness of T cell epitopes contained in two protein sequences and renders results as an EpiCC score (Gutiérrez et al., 2017; Moise et al., 2020). The analysis approach used for the EpiCC analysis is shown in Supplementary Figure 1. Typically, the two input sequences are (a) a vaccine sequence and (b) a field strain sequence. The EpiCC score quantifies the relatedness of the putative epitope content shared between a given pair of sequences. If T cell epitopes in a vaccine closely match the epitopes in the challenge strain, the memory T cells induced by the vaccine are likely to recognize the epitopes in the challenge strain, which will likely facilitate protective immune response. The score of the comparison of T cell epitope content of two sequences (EpiCC score) increases with the presence of additional shared epitopes.

The epitope content of a protein depends on its epitope density, which is defined as the number of epitopes per unit of sequence length and number of MHC alleles. So, if a “high-epitope density” protein is compared to a highly similar protein and many of their epitopes are conserved or shared between the two strains, the EpiCC scores of shared epitopes will be high. Since the PigMatrix binding probabilities of the strain and the vaccine are considered for the calculation, the EpiCC score will be even higher if the shared 9- mer epitopes have high predicted binding probabilities.

EpiCC uses 9-mer sequences and PigMatrix predictions to determine the T cell epitope content shared between a vaccine and a field strain. Nine-mer epitopes are considered shared if they are identical to or if they are “matched” by JanusMatrix (i.e., predicted to bind to the same SLA and exactly matched at the TCR face) (Moise et al., 2013). T cell epitopes with identical TCR- facing residues, which are also predicted to bind to the same MHC allele (cross-conserved epitopes), are considered to be more likely to induce cross- reactive memory T cells. This approach has been used extensively for PCV2 vaccine-strain comparisons and for influenza A vaccine-strain comparisons (Bandrick et al., 2020; Foss et al., 2023; Vargas-Bermudez et al., 2024; Gutiérrez et al., 2017; Tan et al., 2021; Tan et al., 2022).

For each pair of epitopes shared between the vaccine strain and a field strain, the EpiCC algorithm calculates a score based on their joint binding probabilities. Then, all these scores are added to determine the raw EpiCC score for all the epitopes shared between the vaccine and the field strain. This score is normalized by the number of 9-mers in the field strain to express the results in terms of “epitope density,” and by the number of MHC alleles used for T cell epitope prediction, allowing for comparison of EpiCC scores determined using different sets of MHC alleles.

EpiCC scores and coverage analysis (population level and individual pigs): To assess the combined EpiCC scores of the individual proteins of each strain, the raw EpiCC scores of all the epitopes shared between the vaccine and the strain are summed across all the proteins and normalized to the number of 9-mers in the strain, and by the number of MHC alleles used for T cell epitope prediction.

The T cell epitope content of a field strain is considered to be well matched or “covered” by a given vaccine sequence if the strain baseline and the shared EpiCC score are similar. To formally quantify vaccine T cell epitope coverage, the EpiCC score of each vaccine-field strain comparison was divided by that field strain baseline EpiCC score and expressed as a percentage. For the individual pig analysis, the EpiCC scores were calculated using the SLA haplotype of the pigs and calculating the scores for the challenge strain to which they were exposed to.

## Results

### T cell epitope content of PRRSV-2 structural proteins

**FIGURE 1:**
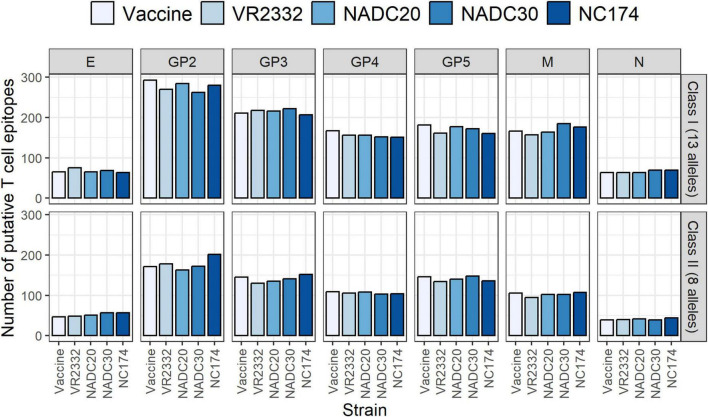
Number of putative T cell epitopes for each strain and for each protein. The absolute number of class I (top) and class II T cell epitopes is shown for the MLV vaccine and each challenge strain, under the protein name. The top histograms show Class I T cell epitope content and the bottom histograms show class II T cell epitope content.

Based on analysis using PigMatrix, in total, the four PRRSV strains contained slightly fewer SLA class I predicted T cell epitopes than the vaccine (Figure 1 and Supplementary Table 1). Overall, E and N had the smallest number of putative epitopes and GP2 the largest. It is worth noting that differences in (a) the number of alleles used for prediction (13 alleles for class I and eight alleles for class II), and (b) the length of the input sequences, has an impact on the number of epitopes predicted. For class II, only NC174 contained more T cell epitopes than the vaccine. NC174’s GP2 had the largest difference in number of predicted class II epitopes compared to the vaccine. Taken together, with respect to overall putative T cell epitope content, there is minimal difference in the total number of epitopes between the vaccine strain and the analyzed strains, although the epitopes may not be identical in sequence.

### Population level EpiCC results

**FIGURE 2:**
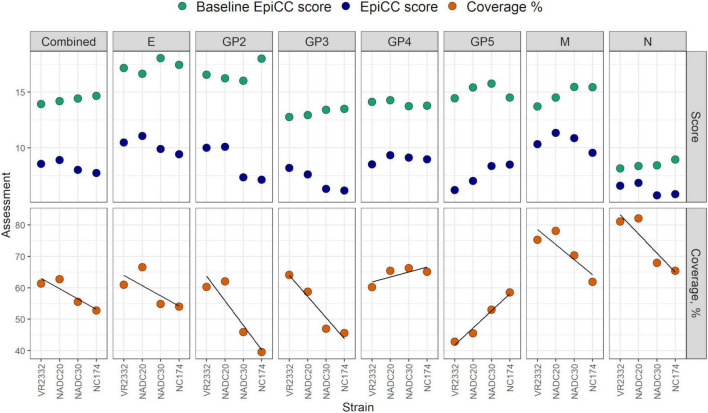
EpiCC scores and T cell epitope coverage. Baseline EpiCC scores, EpiCC scores and T cell epitope coverage percentages calculated for seven individual proteins from four PRRSV-2 strains are shown. Linear regression lines are displayed in each T cell epitope coverage panel.

We compared the putative class I and class II T cell epitope content of the vaccine against that of protein sequences from four PRRSV challenge strains. A summary of our results is presented in Figure 2 and Supplementary Table 2. The average (combined) coverage of the challenge strains by the MLV vaccine protein sequences was 58.09%, ranging from 52.76 (NC174) to 62.72% (NADC20). Based on the combined T cell epitope content of the seven proteins, protective efficacy was anticipated to be highest for NADC20 (62.72%), VR2332 (61.34%), and lower for NADC30 (55.55%), and NC174 (52.76%).

Baseline EpiCC scores: The baseline EpiCC score is a metric representing the T cell epitope content of the sequences in the context of a self-comparison (i.e., T cell epitope content of each sequence compared to itself) and, as such, represents the highest achievable EpiCC score for a sequence. In this SLA class I and II analysis, baseline EpiCC scores are the sum of the individual class I and II SLA baseline EpiCC scores. Overall, the baseline scores ranged from 8.14 (N, VR2332) to 18.06 (E, NADC30), suggesting that the overall T cell epitope density differed among these proteins included in this analysis. E proteins had on average, the highest baselines, while N proteins had the lowest, indicating the highest and lowest total T cell epitope content, respectively. However, within each protein, baseline scores were similar among strains. The combined baseline EpiCC scores, which aggregate the protein-specific baseline EpiCC scores per strain, were also similar, ranging from 13.94 (VR2332) to 14.67 (NC174).

EpiCC scores: A comparative analysis of the T cell epitope content of the vaccine strain with the four PRRSV challenge strains revealed an average combined EpiCC score of 8.3 (range of 7.74 for NC174 to 8.9 for NADC20), indicating the greatest degree of shared T cell epitope content with NADC20. For all four PRRSV strains, the EpiCC scores in descending order were: NADC20, VR2332, NADC30, and NC174, with minimal overall differences in EpiCC scores between these strains. The more obvious difference between the combined EpiCC coverage scores is discussed below.

For individual proteins, the EpiCC score ranged from 5.72 (N, NADC30) to 11.33 (M, NADC20). The highest average EpiCC score was observed for the M proteins (10.52), while the lowest average EpiCC scores were observed for the N proteins (6.25). The low EpiCC scores for N proteins are explained by their low baseline EpiCC scores (i.e., low T cell epitope content density). For proteins with low baseline EpiCC scores, EpiCC scores are low even if the vaccine and the strain share a large percentage of their T cell epitopes.

T cell epitope coverage: For a specific vaccine-strain comparison, coverage is the percentage of the T cell epitope content of the challenge strain (baseline EpiCC score) that is shared with the vaccine strain (EpiCC score). T cell epitope coverage is summarized in Supplementary Table 2.

The average combined coverage across all challenge strains was 58.09%, with individual coverage scores ranging from 52.76 (NC174) to 62.72% (NADC20). Thus, among the challenge strains, the vaccine covers the greatest amount of T cell epitope content predicted in NADC20. For the four PRRSV challenge strains, the combined vaccine T cell epitope coverage in descending order was: NADC20, VR2332, NADC30 and NC174 (E, GP2, M and N produced the same ranking).

For the individual proteins, T cell epitope coverage ranged from 42.89 (GP5) to 81.04% (N) for VR2332, from 45.51 (GP5) to 82.02% (N) for NADC20, 45.92% (GP2) to 70.29% (M) for NADC30, and from 39.56 (GP2) to 65.41% (N) for NC174. The lowest coverage observed for GP2 of NC174 was explained by an elevated baseline (NC174 T cell epitope content) and a low EpiCC score (shared T cell epitope content).

Considering all the challenge strains, the average vaccine coverage was the highest for N (74.1%) and the lowest for GP5 (49.98%). The vaccine covered at least 65% of the T cell epitope content identified in N proteins; however, the N sequences contained the lowest T cell epitope content.

Partial protection induced by the MLV vaccine was associated with higher EpiCC scores and T cell epitope coverage: To evaluate the relationship between vaccine efficacy and T cell epitope content shared between the MLV vaccine and the challenge strains, we categorized the vaccination outcomes from Proctor et al. (2022) based gross pathology, viral shedding, and viremia (Table 2). The MLV vaccine was considered partially protective against VR2332, NADC20, and NADC30 and not protective against NC174. For challenge strains against which the MLV vaccine was partially protective, EpiCC scores and T cell epitope coverages were higher than those of the challenge strain against which the vaccine was not protective, suggesting that partial protection was associated with a higher level of shared T cell epitope content.

**TABLE 2:** Vaccine efficacy results from challenge study from Proctor et al. (2022).

Challenge strain	Lineage	Gross pathology	Shedding	Viremia	Categorical outcome^b^
VR2332	5	Virus induced minimal lung lesions	Virus absent or present at low levels in MOCK and challenge groups	Significant reduction	Partial protection
NADC20	8	Significant reduction^a^	Significant reduction	Significant reduction	Partial protection
NADC30	1	Significant reduction	Significant reduction	Significant reduction	Partial protection
NC174	1	Not significant	Not significant	Not significant	No protection

*^a^*A significant reduction refers to a statistically significant reduction reported in Proctor et al. (2022).

*^b^*The vaccine was considered partially protective if it reduced macroscopic pneumonia, viral shedding and viremia.

With exception of GP4 and GP5, vaccine coverage of NC174 T cell epitopes was the lowest. GP5 was the only protein where NC174 T cell epitope coverage was the highest; with a trendline that suggested an inverse correlation with protective immunity (Figure 2, Coverage %). Due to this inverse correlation, the “combined” differences in coverages among strains appears smaller. The inclusion of GP4 and GP5 in the combined score, as the EpiCC and EpiCC coverage scores are inversely correlated with protection, tends to obscure the potential relationship between the combined scores and protective efficacy.

### Individual-level PigMatrix analysis

The second phase of the study involved an analysis of the relationship between the MLV vaccine efficacy and individualized (per-pig) predicted T cell epitope content identified in seven proteins in a cohort of 58 animals. Based on low-resolution SLA typing and published data, we inferred four-digit SLA-DRB1 alleles for seven animals which were among those with low or high CD4 IFN-γ responses that had been measured at 14 DPC. For the EpiCC analyses, PigMatrix prediction models were available for some of the alleles, and T cell epitope prediction models were developed for others.

Individual PigMatrix results: For each animal, 16 input sequences (seven structural proteins plus the ectodomain of GP5 from the vaccine and seven structural proteins plus the ectodomain of GP5 from the challenge strain) were parsed into individual 9-mer frames. Each frame was then assessed for SLA binding potential with respect to the individual animal-specific SLA-DRB1 allele(s). In total, we performed 61,825 frame-by-allele assessments. 491 putative SLA-DRB1 T cell epitopes were identified in the input sequences. The results of the analysis are shown in Table 3.

**TABLE 3:** Number of putative individualized SLA-DRB1-restricted T cell epitopes.

Animal id	Challenge virus	CD4 IFN-γ response (14 DPC)^a^	Inferred SLA-DRB1	Combined^b^	E (73aa)^c^	GP2 (256aa)	GP3 (254aa)	GP4 (178aa)	GP5 (200aa)	GP5 ecto (77aa)	M (174aa)	N (123aa)
B15	NADC20	high	02:01, 07:01	180	15	35	23	29	38	14	29	11
B8	NADC20	high	02:01	99	8	20	13	15	20	8	18	5
G6	VR2332	low	05:01	106	9	27	16	17	19	10	13	5
O19	NC174	low	02:01	106	10	25	15	14	20	8	18	4

*^a^*Response level was defined based on the % IFN-γ+ within CD4 T cells.

*^b^*Total number of putative T cell epitopes identified in the analyzed proteins per strain, excluding GP5 ectodomain.

*^c^*Length of the input protein sequence.

These results demonstrate that the E and N proteins had the least number of putative epitopes and the GP2 protein had the most epitopes. The ectodomain of GP5 contained between 8 and 14 putative SLA-DRB1 T cell epitopes. It is worth noting that differences in (a) the number of alleles used for prediction (two alleles for animal B15 vs. one allele for the other three animals, due to homozygosity), and (b) the length of the input sequences, had an impact on the number of epitopes predicted. Homozygosity, observed in three animals in this study, is believed to be associated with diminished protection against infectious disease; thus, it is reasonable to expect lower EpiCC scores and EpiCC coverage. Overall, the numbers of SLA-DRB1-restricted T cell epitopes were slightly different among animals; the highest combined number of epitopes was observed for the heterozygous animal.

### Individual-level EpiCC results

**FIGURE 3:**
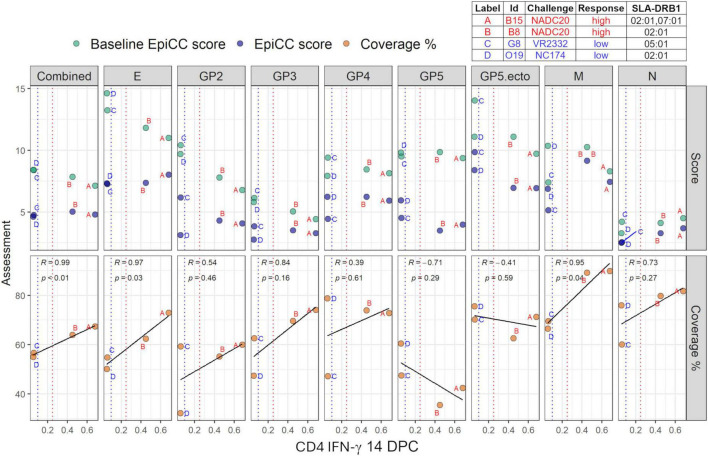
EpiCC scores and T cell epitope coverage. Baseline EpiCC scores (top; green), EpiCC scores (top; blue) and T cell epitope coverage percentages (bottom) calculated for seven individual proteins and the ectodomain of GP5 for high (A, B) and low (C, D) responders are shown. Pearson correlation coefficients, *p*-values, and linear regression lines are displayed in each panel. Dashed vertical lines are the thresholds applied to define low (blue) and high (red) CD4 IFN-γ 14 DPC responses (% IFN-γ+ within CD4 T cells).

The putative animal-specific SLA-DRB1 T cell epitope content of the vaccine was compared to that of protein sequences from PRRSV challenge strains. A summary of the results is presented in Figure 3 and Supplementary Table 3.

Baseline EpiCC scores: As described above, baseline EpiCC scores represent the T cell epitope content of the challenge strain sequences following a self-comparison. The baseline scores ranged from 3.29 (N) to 14.62 (E) for the animal challenged with NC174 (pig O19), indicating differences in T cell epitope densities among proteins. E proteins had the highest average baselines and N proteins had the lowest, indicating the lowest total T cell epitope content. Even though low responders had SLA alleles that presented slightly higher T cell epitope densities, the epitopes they presented were not as conserved between vaccine and challenge strain as they were in protected pigs, as will be shown below. The key difference between high and low protection may relate to the number of epitopes that are conserved between the vaccine and the challenge strain.

EpiCC scores: The combined EpiCC scores ranged from 4.63 (NC174, pig O19; low responder) to 5.03 (NADC20, pig B8; high responder). For these four animals, EpiCC scores were slightly higher for high responders, both challenged with NADC20. The differences in scores for both pigs challenged with NADC20 are explained by differences in the putative T cell epitope content restricted by animal-specific SLA-DRB1 alleles (and homozygosity). Since both animals expressed SLA-DRB1*02:01, the differences may be driven by SLA-DRB1*07:01, expressed by pig B15.

For individual proteins, the EpiCC score ranged from 2.5 (N, pig O19; low responder) to 9.87 (GP5 ectodomain, G6; low responder). N had, the lowest average EpiCC score. The low EpiCC scores for N proteins are explained by their low baseline EpiCC scores (i.e., low T cell epitope content density).

EpiCC coverage scores: Higher EpiCC coverage scores, per individual pig, appear to be associated with protection for this small cohort. Thus for protection from specific strains of PRRSV—higher protection can be expected when more T cell epitopes match the vaccine and the genetic background of the pig (SLA haplotype). This individual-level evaluation of pigs for which SLA typing and prediction models were available, shows that some SLA haplotypes present more and some SLA haplotypes present less T cell epitopes from the vaccine. Thus, T cell-epitope driven protection against challenge varied based on SLA haplotype for a given pig, vaccine, and challenge strain combination.

The combined coverage ranged from 55.03 (O19; low responder) to 67.38% (B15; high responder, Supplementary Table 3). The combined T cell epitope coverage was higher for high responders than for low responders. CD4 IFN-γ responses measured at 14 DPC were significantly correlated (*p* < 0.01) with the combined animal-specific SLA-DRB1 T cell epitope coverage (Figure 3, left bottom panel).

For the individual proteins, and consistent with the combined T cell epitope coverage, coverage was higher for high responders for E, GP3, M, and N. For GP2, T cell epitope coverage was similar between the two high responders and the low responder challenged with VR2332 (G6). For GP4, T cell epitope coverage was the highest for the low responder challenged with NC174 (O19). For GP5, coverage was higher for low responders than for high responders. A simple threshold cannot be clearly defined, but in general, partial protection was observed for those animals with SLA alleles that could present more than 58% of most of the proteins’ epitopes that were conserved between the vaccine and the challenge strain. Except for GP5 and its ectodomain, the linear relationship between CD4 IFN-γ and T cell epitope coverage were positive for each of the proteins. Correlations for CD4 IFN-γ and T cell epitope coverage for E and M were significant.

For GP5, T cell epitope coverage was generally higher for low responders than for high responders. The negative relationship observed for GP5 suggests that GP5’s predicted T cell epitope content shared between vaccine and challenge strains does not have a direct relationship with CD4 IFN-γ responses. However, the total number of pigs that were evaluated in this cohort may be too small to make a strong correlation. Further SLA typing would improve the accuracy of this finding.

## Discussion

### Population level pig-vaccine-challenge analysis

A comparative analysis was conducted on the T cell epitope content of seven proteins (E, GP2, GP3, GP4, GP5, M, and N) from the MLV vaccine and four wild-type PRRSV strains (VR2332, NADC20, NADC30, and NC174). To complete this analysis, we performed individual comparisons between the vaccine and the strains using our EpiCC algorithm. We also calculated the baseline T cell epitope content of each strain and the percentage of the strain’s baseline that was covered by the vaccine. Our analysis was focused on class II T cell epitopes (CD4+) due to the observation that CD4+ T cell responses were correlated with protective efficacy in the publication (Proctor et al., 2022) analyzed here.

Considering the combined T cell epitope content of all the analyzed proteins, putative MLV vaccine T cell epitopes shared with the PRRSV strains covered on average 58.09% of their total epitope content, ranging from 52.76 (NC174) to 62.72% (NADC20). While cross-reactive responses are expected at this level of coverage, they might be limited. Although we are unaware of a specific coverage threshold that may confer complete or partial protection, we believe that higher T cell epitope coverage would likely result in stronger cross-reactive cell-mediated immune responses. For these four PRRSV strains and based on the combined T cell epitope content of seven proteins, the strains against which the MLV vaccine may confer broader cross-reactive cell-mediated immune response are: NADC20 (62.72%), VR2332 (61.34%), NADC30 (55.55%), and NC174 (52.76%). However, the small differences in T cell epitope coverage between NADC20 and VR2332 and NADC30 and NC174 might not produce measurable differences experimentally. Further validation studies are planned to better understand these differences.

It is worth noting that GP3, GP4, and GP5 produced different rankings based on T cell epitope coverage. Vaccine coverage rankings for individual animals expressing different SLA alleles also differed. Thus far, the results of GP5 seemed to be inversely correlated with the protective efficacy of the MLV vaccine. These results suggest that while current genotyping depends on GP5 analysis, it is unlikely that the genotyping performed using GP5 will be closely associated with protective relationships between vaccines and lineages. These results indicate a whole genome analysis may provide a better understanding a broad neutralizing immune response to PRRSV.

This initial assessment also suggests that immune responses to E, GP2, M, and N, are more likely to be associated with protective immunity. With additional detailed information from SLA typed pigs, we might be able to determine whether class I or II EpiCC results of a specific protein or set of alleles correlate better with CD4, CD8 T cell responses and complete or partial protection. The results of the correlation analysis might improve our ability to predict whether a vaccine would induce cell-mediated immune responses, protection, or partial protection. Moreover, should further studies determine that either cross- reactive class I or II epitopes are more relevant for protection with this vaccine, a weighted EpiCC score that favors class I or II epitopes could be applied.

Based on the combined T cell epitope content of seven proteins for these PRRSV-2 strains, broader cross-reactive cell-mediated immune response was anticipated to be higher for the NADC20 and VR2332 vaccines and lower for the NADC30 and NC174 vaccines. These results were consistent and correlated with the published vaccine efficacy data (Proctor et al., 2022). The protection that was induced by the MLV vaccine was associated with higher EpiCC scores and T cell epitope coverage.

In summary, the findings of the present study suggest that elevated EpiCC scores and the extent to which challenge strain epitopes are covered by the vaccine are associated with a greater degree of protection against challenge strains within the population.

### Individual pig-vaccine-challenge analysis

To complete our analysis, we performed individual comparisons between the vaccine and challenge strains. EpiCC was performed using animal-specific SLA-DRB1 alleles for four animals, two low responders and two high responders based on CD4 IFN-γ production measured at 14 DPC. For this small subset of four animals, CD4 IFN-γ responses were significantly correlated with animal-specific SLA-DRB1-restricted T cell epitope coverage. The combined T cell epitope coverage was higher for high responders than for low responders. For E, GP3, M and N, T cell epitope coverage was also higher for high responders. The correlations between CD4 IFN-γ responses and T cell epitope coverage was significant for E and M. Only GP5 and its ectodomain had negative correlations, suggesting that the T cell epitope content shared between the MLV vaccine and the challenge strains was not directly correlated with CD4 IFN-γ responses for those proteins.

EpiCC compares T cell epitopes rather than B cell epitopes. Thus the inverse correlation between GP5 and protective efficacy would not be due to the presence of “decoy” epitopes (which are targeted by antibodies). We have previously evaluated Pertussis, HIV, and influenza surface proteins and have found that these surface proteins trend lower, in terms of T cell epitope content, and may contain Treg epitopes. Future anticipated studies of T cell responses to GP5 epitopes may help determine the relationship between these aspects of the adaptive immune responses and the protective efficacy of PRRSV vaccines.

In general, higher T cell epitope conservation between vaccines and challenge strains appears to have a positive effect on protection. Considering the combined T cell epitope content of all the analyzed proteins, putative MLV vaccine T cell epitopes shared with the PRRSV strains covered between 55.03 (NC174; low responder) to 67.38% (NADC20; high responder). While some CD4 IFN-γ responses were observed at this level of coverage, it is reasonable to believe that higher T cell epitope coverage would likely result in stronger cross-reactive cell-mediated immune responses.

The EpiCC data of individual animals recapitulates population-level T cell epitope coverage results. T cell epitope coverage for the larger cohort indicated that greater than 58% coverage of challenge strain epitopes (for the combined proteins) by the vaccine might be associated with a degree of protection from disease. This finding is supported by the study of individual animals, in which a combined T cell epitope coverage above 58% again appeared to be associated with protective efficacy for this small subset of four animals.

Both high responders included in the analysis were challenged with NADC20. Analyzing high responders challenged with NC714 and NADC30 and low responders challenged with NADC20 (Table 1) could provide additional information to better assess the relationship between IFN-γ responses and animal-specific SLA-DRB1-restricted epitope content shared between MLV vaccine and the challenge strains. EpiCC data generated for animals with intermediate responses might also contribute to a more comprehensive understanding of this relationship. In addition, the availability of class I and II high-resolution SLA typing data could expand our analysis to other animal-specific metrics of vaccine efficacy.

### Additional considerations

This analysis did not account for PRRSV ability to alter immune responses (Hernández et al., 2021; Loving et al., 2015) or the presence of potential regulatory T cell (Treg) epitopes. In a preliminary analysis of the submitted sequences using JanusMatrix adapted for swine, clusters of putative T cell epitopes were identified, that were cross-conserved with predicted epitopes derived from multiple proteins from the swine proteome. In humans, peptides highly cross-conserved with human proteins can either be tolerated or potentially be actively regulatory (Jang et al., 2020; Moise et al., 2015). The presence of Treg epitopes is another factor that might alter the relationship between the vaccine and the strains if some of the vaccine T cell epitopes are regulatory, and those are either present or absent in the strains, or vice versa. To identify putative T cell epitopes for designing a vaccine, it may be important to define the Treg epitopes that may be present in PRRSV, and to perform a detailed analysis of the results that incorporates an adjustment for potential Treg epitopes.

Another variable not considered in this analysis is the difference in virulence between the analyzed strains. Furthermore, the calculation of combined scores was based on the summation of the T cell epitope content of each individual protein, under the assumption of identical levels of expression among the proteins. Future models could explore the potential effects on shared T cell epitope content and coverage considering different levels of expression for individual proteins.

The analysis did not include non-structural proteins (NSP). These proteins may contribute to immunosuppression (Huang et al., 2015; Sun et al., 2010), thus it may be important to evaluate the ability of vaccines to drive immune responses to these NSP proteins. However, as compared to structural proteins, non-structural proteins (NSP) are well conserved. Because of their amino acid sequence similarity, limited T cell epitope variability is expected among non-structural proteins, and it is unlikely that EpiCC analysis would demonstrate significant differences related to the vaccination efficacy.

Finally, the analysis did not make a comparison between vaccines. Rather, the study focused on a single vaccine against four strains. Vaccine-to-vaccine efficacy comparisons, particularly with different MLV vaccines, may need to consider additional factors besides T-cell responses such as innate and cellular immune responses (e.g., NK cells), as well as antibody responses. Some vaccines may induce higher levels of innate immune cytokines, resulting in greater B cell response or presentation of T cell epitopes. In turn, this would have an impact on efficacy outside of a strict T cell epitope comparison.

## Conclusion

These sets of data provide strong support for the role of T cell-mediated responses in PRRSV and should encourage vaccine developers to consider T cell epitope content when developing new vaccines. Livestock producers may also wish to focus on identifying the SLA background of their herds, as some SLA combinations may render pigs more susceptible to disease after PRRSV infection and may be associated with lower protection from selected vaccines.

## Data Availability

The original contributions presented in this study are included in this article/Supplementary material, further inquiries can be directed to the corresponding author.
