# Supplementary material for: T cell epitope content comparison using EpiCC correlates with vaccine efficacy against heterologous porcine reproductive and respiratory syndrome virus type 2 strains
Source: Front Microbiol. 2025 Jun 26;16:1625309. doi: 10.3389/fmicb.2025.1625309 (PMC12241073; doi:10.3389/fmicb.2025.1625309)
Supplement: Supplementary file 1 [file Data_Sheet_1.pdf]

Supplementary Material

1 Supplementary Figures and Tables

1.1 Supplementary Figures

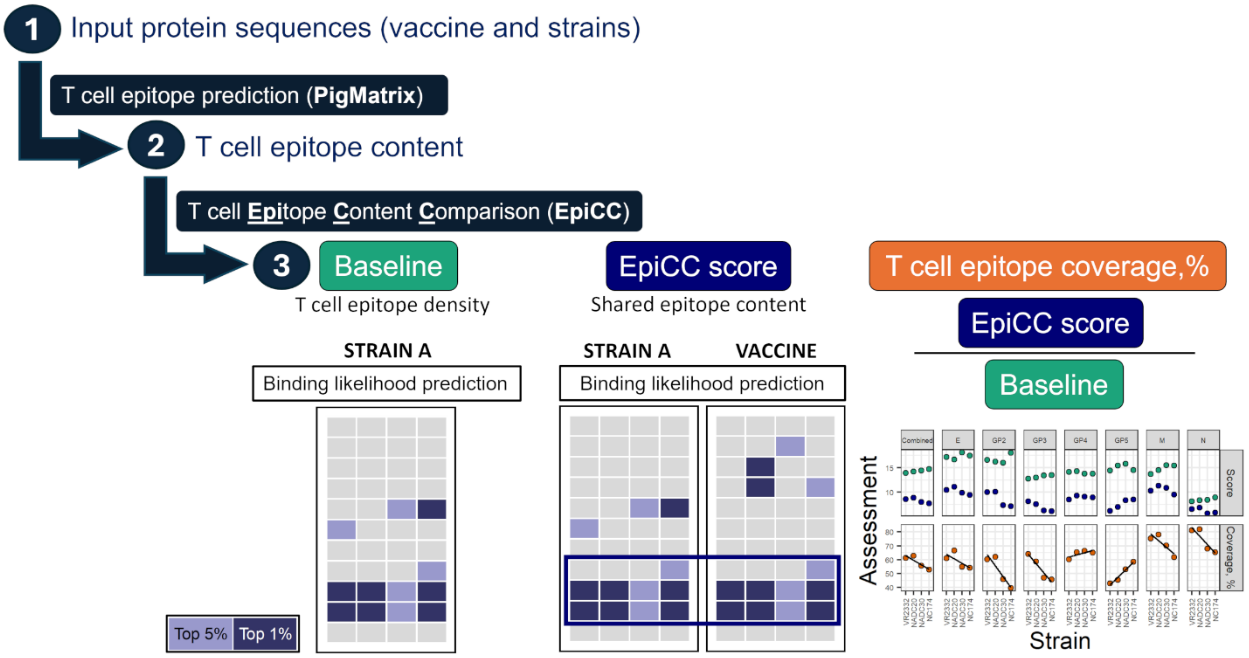

Supplementary Figure 1. EpiCC analysis approach.

1.2 Supplementary Tables

Supplementary Table 1. Number of putative T cell epitopes

Alleles	Strain	Total[a]	E (73 aa)[b]	GP2 (256 aa)	GP3 (254 aa)	GP4 (178 aa)	GP5 (200 aa)	M (174 aa)	N (123 aa)
Class I (13 alleles)	**Vaccine**	**1,146**	**65**	**293**	**211**	**167**	**181**	**166**	**63**
	VR2332	1,100	75	270	218	156	161	157	63
	NADC20	1,125	65	284	216	156	177	164	63
	NADC30	1,130	68	262	222	152	172	185	69
	NC174	1,106	63	280	207	151	160	176	69
	**Sum**	**5,607**							
Class II (8 alleles)	**Vaccine**	**763**	**47**	**171**	**145**	**109**	**146**	**106**	**39**
	VR2332	731	48	178	130	106	134	95	40
	NADC20	741	51	163	135	108	140	102	42
	NADC30	762	57	172	141	103	148	102	39
	NC174	802	57	202	152	104	136	107	44
	**Sum**	**3,799**							

[a]Total number of putative T cell epitopes identified in the analyzed proteins per strain.
[b]Length of the input protein sequence.

Supplementary Table 2. Summary of EpiCC scores and T cell epitope coverage (population-level)

Assessment	Strain	Combined[a]	E (73 aa)	GP2 (256 aa)	GP3 (254 aa)	GP4 (178 aa)	GP5 (200 aa)	M (174 aa)	N (123 aa)
Baseline EpiCC score	VR2332	13.94	17.18	16.57	12.76	14.13	14.45	13.71	8.14
	NADC20	14.19	16.64	16.25	12.94	14.27	15.42	14.52	8.35
	NADC30	14.43	18.06	16.02	13.40	13.75	15.76	15.47	8.42
	NC174	14.67	17.45	18.00	13.50	13.77	14.50	15.44	8.94
	Average	14.31	17.33	16.71	13.15	13.98	15.03	14.79	8.46
EpiCC score	VR2332	8.55	10.47	9.99	8.18	8.50	6.20	10.32	6.60
	NADC20	8.90	11.07	10.09	7.61	9.33	7.02	11.33	6.85
	NADC30	8.02	9.90	7.36	6.30	9.11	8.36	10.87	5.72
	NC174	7.74	9.43	7.12	6.16	8.97	8.48	9.55	5.85
	Average	8.30	10.22	8.64	7.06	8.98	7.51	10.52	6.25
T cell epitope coverage, %	VR2332	61.34	60.97	60.28	64.12	60.16	42.89	75.25	81.04
	NADC20	62.72	66.51	62.07	58.77	65.40	45.51	78.06	82.02
	NADC30	55.55	54.83	45.92	47.02	66.27	53.03	70.29	67.93
	NC174	52.76	54.02	39.56	45.64	65.13	58.48	61.85	65.41
	Average	58.09	59.08	51.96	53.89	64.24	49.98	71.36	74.10

[a]Combined scores calculated by combining the Class I- and II-restricted T cell epitope content of the seven analyzed proteins.

Supplementary Table 3. Summary of EpiCC scores and T cell epitope coverage (individual-level)

Assessment	Animal id	Challenge virus	CD4 IFN-γ 14 DPC[a]	Response	Inferred SLA-DRB1	Combined[b]	E (73aa)	GP2 (256aa)	GP3 (254aa)	GP4 (178aa)	GP5 (200aa)	GP5 ecto (77aa)	M (174aa)	N (123aa)
Baseline EpiCC score	B15	NADC20	0.68	high	02:01,07:01	7.14	11.01	6.80	4.44	8.15	9.38	9.73	8.31	4.52
	B8	NADC20	0.45	high	02:01	7.87	11.82	7.80	5.06	8.45	9.86	11.10	10.28	4.13
	G6	VR2332	0.059	low	05:01	8.41	13.25	10.42	6.16	9.41	9.52	14.04	7.42	4.23
	O19	NC174	0.055	low	02:01	8.42	14.62	9.70	5.82	7.93	9.83	11.10	10.36	3.29
EpiCC score	B15	NADC20	0.68	high	02:01,07:01	4.81	8.03	4.08	3.29	5.94	3.98	6.95	7.46	3.69
	B8	NADC20	0.45	high	02:01	5.03	7.38	4.31	3.52	6.25	3.50	6.96	9.16	3.29
	G6	VR2332	0.059	low	05:01	4.77	7.27	6.18	3.86	4.45	4.53	9.87	5.16	2.54
	O19	NC174	0.055	low	02:01	4.63	7.34	3.13	2.76	6.25	5.95	8.40	6.90	2.50
T cell epitope coverage, %	B15	NADC20	0.68	high	02:01,07:01	67.38	72.99	59.98	74.11	72.86	42.43	71.36	89.81	81.74
	B8	NADC20	0.45	high	02:01	63.96	62.43	55.26	69.63	73.96	35.51	62.68	89.14	79.79
	G6	VR2332	0.059	low	05:01	56.69	54.86	59.33	62.64	47.28	47.57	70.28	69.52	60.06
	O19	NC174	0.055	low	02:01	55.03	50.17	32.29	47.42	78.85	60.54	75.63	66.53	76.03

[a]CD4 IFN-γ 14 DPC responses measured as % IFN-γ+ within CD4 T cells.

[b]Combined scores calculated by combining the SLA-DRB1-restricted T cell epitope content of the seven analyzed proteins (excluding the ectodomain of GP5).
